# Automated Global Positioning Layout *(GPL)* for accuracy assessment in CAD-CAM mandibular reconstruction – Method validation

**DOI:** 10.1038/s41598-025-30516-1

**Published:** 2026-02-25

**Authors:** Elisa Vargiu, Laura Tognin, Giordana Bettini, Giorgia Menapace, Piero Franco, Giorgia Saia, Giorgio Bedogni, Roberto Meneghello, Alberto Bedogni

**Affiliations:** 1 Department of Management and Engineering, University of Padua, Padua, Italy; 2 Maxillo-Facial Surgery Unit, Head and Neck Department, University Hospital of Parma, Parma, Italy; 3 Maxillofacial Surgery Unit, ‘‘S. Anna’’ Hospital, Como, Italy; 4 Department of Clinical Orthopaedics, University of Florence, A.O.U Careggi CTO Florence, Florence, Italy; 5 Department of Neuroscience, Unit of Maxillofacial Surgery, University of Padua, Padua, Italy; 6 Department of Medical and Surgical Sciences, Alma Mater Studiorum- University of Bologna, Bologna, Italy; 7 Department of Primary Health Care, Internal Medicine Unit addressed to Frailty and Aging, “S. Maria delle Croci” Hospital, AUSL Romagna, Ravenna, Italy; 8 Regional Center for the Prevention, Diagnosis, and Treatment of Medication and Radiation-related Bone Diseases of the Head and Neck, Hospital Trust of Padova, Padova, Italy

**Keywords:** Mandibular reconstruction, Computer-Aided Design,, Computer-Aided Manufacturing,, Prosthesis,, Accuracy assessment, Validation, Computational biology and bioinformatics, Engineering, Mathematics and computing, Medical research

## Abstract

The lack of a standardized methodology complicates accuracy assessment in computer-assisted mandibular reconstruction. Existing landmark-based methods are susceptible to operator variability, while surface-based comparisons can mask local deviations. This study validates a novel, automated protocol, the Global Positioning Layout (GPL), to quantify the 3D discrepancy between the virtual surgical plan and the postoperative outcome, by comparing its performance and reliability against Methods A and B. A retrospective cohort of 17 patients was analysed, with three operators performing all measurements on two occasions. The GPL method demonstrated complete reproducibility, with no inter- or intra-operator variability, providing a detailed, spatially-oriented assessment of deviations. In contrast, the landmark-based method showed poor reproducibility and systematic bias and was often inapplicable due to the absence of landmarks after resection. The surface-based method, while objective for its mean error metric, was operator-dependent for initial alignment and its non-directional output masked significant localized deviations. This study validates GPL as a robust and fully reproducible tool that overcomes the critical limitations of established techniques. The GPL method provides a strong foundation for a standardized protocol, essential for the reliable comparison of surgical outcomes, refinement of surgical techniques, and improvement of long-term patient outcomes.

**Supplementary Information:**

The online version contains supplementary material available at 10.1038/s41598-025-30516-1.

## Introduction

The use of computer-aided design and manufacturing (CAD/CAM) has become integral to head and neck surgery, allowing for the creation of patient-specific solutions to restore facial aesthetics and function. By providing detailed 3D visualizations, virtual surgical planning (VSP) has significantly improved the precision of both surgical planning and execution. When combined with computer-assisted surgery (CAS), these technologies improve surgical accuracy and provide the consistent, objective data necessary for the robust evaluation of clinical outcomes^1,2^.

Recent systematic reviews and efforts to create evaluation guidelines have underscored a lack of methodological standardization in accuracy assessment^3^. A variety of approaches have been reported in the literature, many of which have been developed for and partly validated in the context of reconstructions using microvascular free bone flaps. These methods generally rely on linear or angular measurements derived from specific anatomical landmarks, but this approach often depends on manual point selection, introducing potential intra- and inter-operator variability^4–14^. Alternatively, three-dimensional (3D) surface comparison techniques provide a more global assessment of congruence, though their interpretation can be challenging and they may lack specific 3D spatial orientation information^9,15,16^. Another approach provides a comprehensive assessment by superimposing 3D models to generate a roto-translational matrix that numerically describes the spatial deviation, although a common limitation of this method is the lack of a standardized reference system^17^.

To address these limitations, the Global Positioning Layout (GPL) method was developed to standardize the quantification of three-dimensional spatial discrepancies between planned reconstructions and postoperative outcomes, eliminating operator dependency^18^. The present study aims to validate the GPL method by comparing its performance against two established protocols from the literature that utilize manual, linear measurements^8^ and global, three-dimensional surface analysis^16^.

## Materials and methods

### Patient population

A consecutive series of patients who underwent mandibular reconstruction with custom-made prosthetic devices (REPLICA) at the Maxillofacial Surgery Unit, University Hospital of Padova, Italy^19^ was retrospectively collected for validation. Of the 18 patients operated between March 2012 and June 2017, one who died from postoperative complications was excluded. The final series comprised 17 patients (9 females, 8 males) with a median age of 67 years (IQR 65–73). The study was conducted in accordance with the Declaration of Helsinki, and the protocol was approved by the Ethical Committee of the University Hospital of Padova (protocol number 24435/AOP1814 April 2019); all patients gave their written informed consent. Detailed patient characteristics, including demographics, primary diagnoses and defect classification^20^ are summarized in Table 1.

**Table 1:** Demographics, clinical characteristics, and mandibular defect classification of the study cohort.

Patient characteristics	*N* = 17
*Sex*:	
Woman	9
Man	8
*Age (years)*:	67 (65;73)
*Race*:	
Caucasian	17
*Operational diagnosis*:	
Ameloblastoma	2
Medication-related osteonecrosis of the jaw	7
Osteoradionecrosis	2
Squamous cell carcinoma of the oral cavity	2
Mandibular reconstruction plate failure	3
Chronic osteomyelitis	1
*Underlying disease*:	
Metastatic breast cancer	5
Personal history of orofaringeal cancer (SCC)	1
Multiple myeloma	2
None	4
Ossifying fibroma	1
Metastatic prostate cancer	1
Squamous cell carcinoma of the oral cavity	2
Radiodermatitis (II grade)	1
*Mandibular defect (Boyd et al.*,* 1993 [20])*:	
LCL left	1
L right	1
L left	1
HCL right	2
HCL left	1
HC left	1
H right	3
H left	7

### Preparation and virtual workflow for accuracy assessment

Each patient underwent preoperative volumetric or thin-slice computed tomography (CT) scanning, with the acquired data stored in DICOM format. Using Mimics software (Materialise, NV, Leuven, Belgium), the segmentation process was performed to isolate regions of interest, and Virtual Surgical Planning (VSP) was conducted using Geomagic Freeform software (3D Systems, Inc., South Carolina, USA), to establish the surgical plan and generate the preoperative 3D models. To evaluate the accuracy of the surgical outcome against the virtual plan, operated patients underwent a thin-slice CT scan one month postoperatively, according to a standard follow-up protocol. The postoperative DICOM data were then segmented to generate the corresponding postoperative model. A detailed description of the methodology used to generate the specific virtual models has been recently described^18^.

### Accuracy assessment methodologies

To validate the Global Positioning Layout^18^ method, its performance was compared against two established methodologies selected from the literature to represent different underlying principles:

Method A^8^, a manual, landmark-based protocol using linear distance measurements, and Method B^16^, a semi-automated, surface-based technique that assesses global model congruence via the Hausdorff distance. Each protocol is detailed in the following subsections. ^1816^

### Global positioning layout

**Fig. 1:**
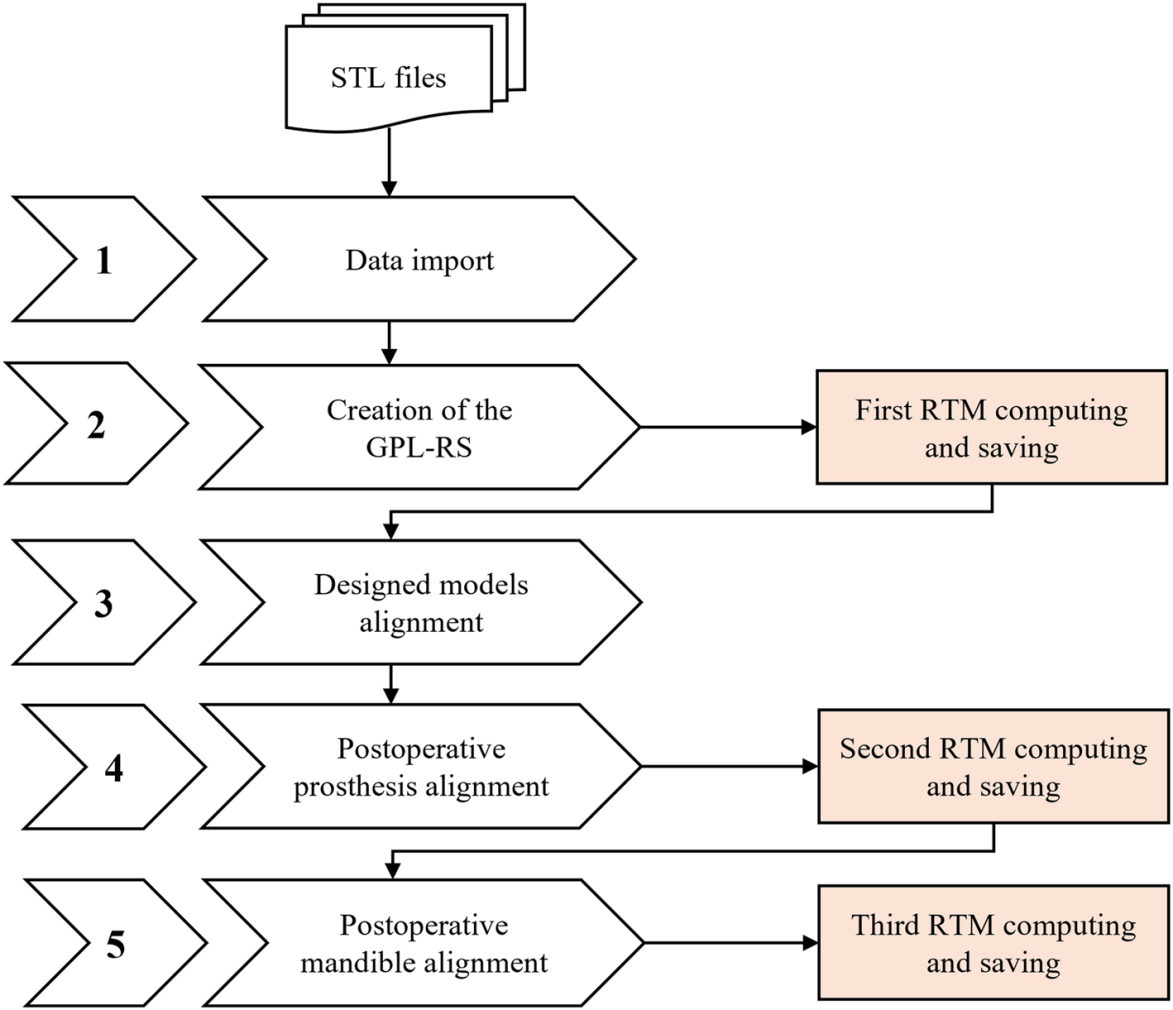
Schematic flowchart of the Global Positioning Layout (GPL) workflow.

Following the methodology previously described by our group^18^, the GPL approach quantifies reconstruction accuracy via roto-translational matrices (RTMs) representing the 3D spatial deviations between planned and postoperative outcomes. A key feature is the establishment of a unique coordinate reference system (GPL-RS) based on the ‘reference mandible’ geometry, providing a stable and objective framework for comparison. The definition of the GPL-RS establishes an initial RTM which is used to align the planned mandible and the designed prosthesis within this reference system. Subsequently, the postoperative prosthesis (floating model) is registered to the designed prosthesis (fixed model) using an Iterative Closest Point (ICP) algorithm. This second RTM, obtained from prosthesis registration, is then applied to the postoperative mandible component, ensuring that both the designed and postoperative models are located within the GPL-RS. The third and final RTM, derived from aligning the postoperative mandible to the planned mandible, quantifies accuracy in terms of rotational and translational reconstruction errors. The methodological workflow is summarized in Fig. 1.

### Method A

**Fig. 2:**
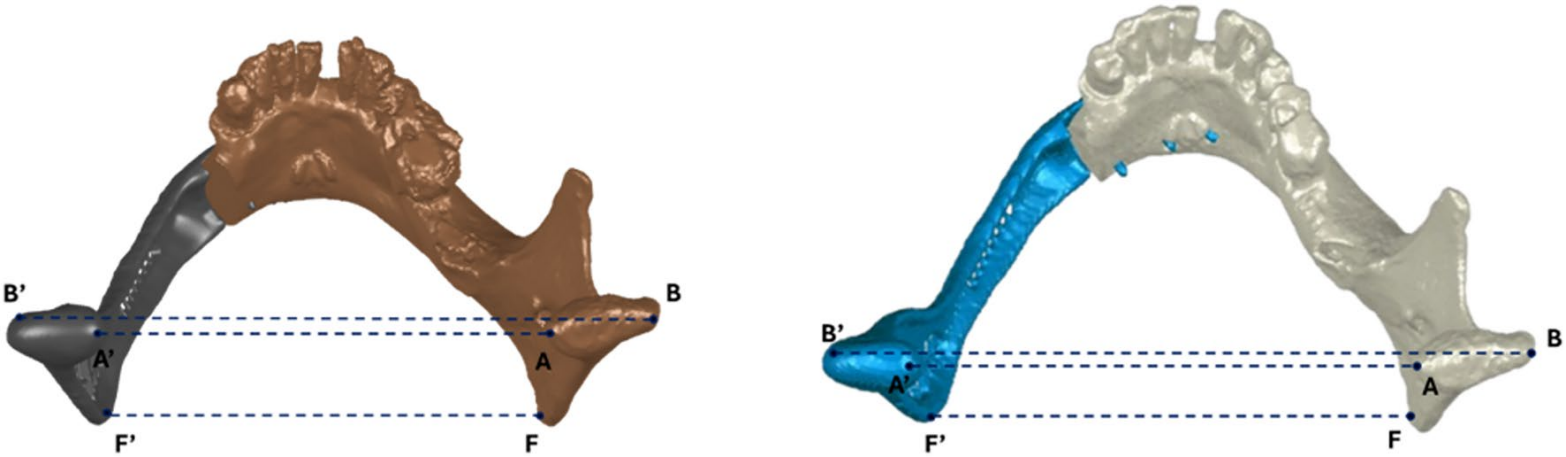
Selected landmarks for a type H defect in the planned model (left) and the postoperative model (right).

Initially described by Wilde et al.^8^, this method defines mandibular reconstruction accuracy by measuring the postoperative transverse deviation of the mandibular rami from their preoperative position. Six corresponding landmark pairs are identified on both the virtual surgical planning (VSP) and postoperative models, where anatomically feasible: A–A′ (*innermost point of right mandibular condyle to innermost point of left mandibular condyle*,), B–B′ (*outermost point of right mandibular condyle to outermost point of left mandibular condyle*), C–C′ (*lowest point of right mandibular notch to lowest point of left mandibular notch*), D–D′ (*tip of right lingula of the mandible to tip of left lingula of the mandible*), E–E′ (*tip of right coronoid process to tip of left coronoid process*), and F–F′ (*most caudal point of right mandibular angle to most caudal point of left mandibular angle*). In our 17-case cohort, A–A′, B–B′, and F–F′ were consistently measurable in all patients (Fig. 2), whereas C–C′ and E–E′ could be reliably identified in only two subjects due to specific anatomical feature. For each identified landmark pair, the distance between corresponding points was measured three times on both models using the *Measure function* in Geomagic Wrap^®^ (Oqton, 3D Systems, Inc., South Carolina, USA). The mean of these three measurements was calculated to determine the average VSP and postoperative distances for each pair. Accuracy was then determined using the Eq. 1:

1 $$\:Accuracy\:=\:\left(mean\:postoperative\:distance\right)\:-\:\left(mean\:VSP\:distance\right)$$

### Method B

This evaluation method described by Tarsitano et al.^16^ leverages the three-dimensional analysis.

**Fig. 3:**
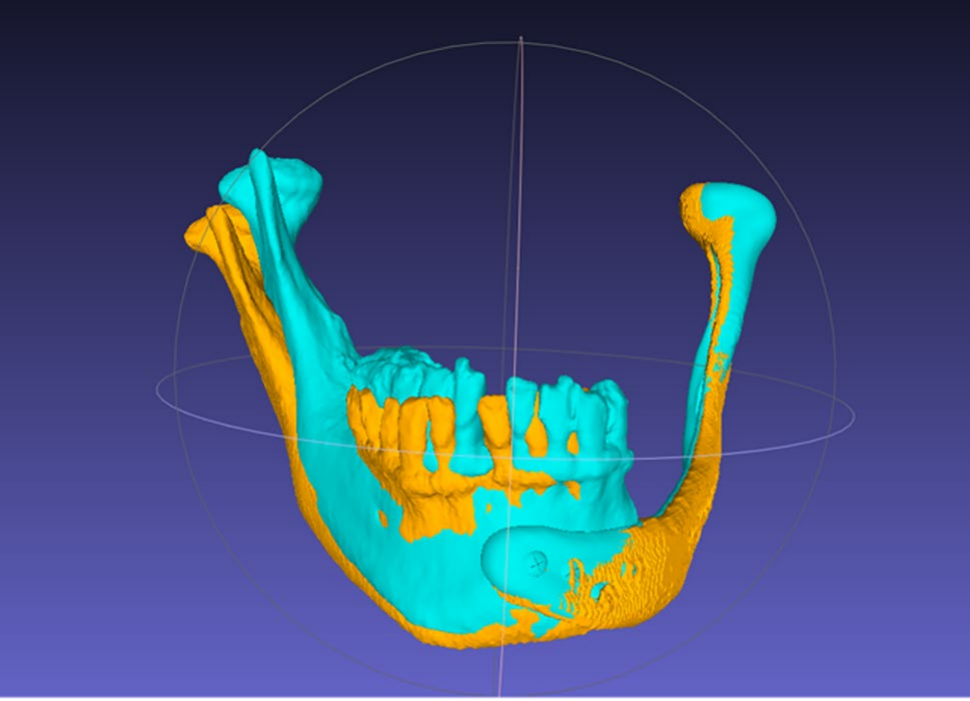
Superimposition of the two 3D virtual models using the Align function of the MeshLab software. VSP model (light blue) and postoperative model (orange).

Initially, the virtual surgical planning (VSP) and postoperative models are imported into the open-source software MeshLab (Visual Computing Lab, ISTI-CNR, Pisa, Italy). A semi-automated alignment is then performed (Fig. 3): the operator manually selects at least four corresponding points on both models to obtain an initial registration, which the software automatically refines to complete the surface superimposition. Once aligned, the Hausdorff distance function is used to calculate the final accuracy metrics - minimum, maximum, and mean Hausdorff distances (± RMS) - for each patient.

### Statistical analysis

To evaluate the accuracy and reliability of all three assessment methods (GPL, Method A, and Method B), each protocol was performed by three independent operators (O1, O2, O3) on two separate occasions. A Linear Mixed-Effects Model (LMEM) was subsequently employed for the statistical analysis. This approach was chosen for its ability to properly handle repeated measurements clustered within patients, thus accounting for the correlation between observations. The model was specified to evaluate the impact of the operator, the measurement occasion, and their interaction as fixed effects on the outcome variable. To account for subject-specific variability, a random intercept was included for each patient. From the fitted model, estimated marginal means and 95% confidence intervals were calculated, and pairwise comparisons were performed to quantify both intra- and inter-operator variability.

## Results

### Global positioning layout (GPL) analysis

The accuracy results obtained with the GPL method are presented in Table 2. Regarding the rotational components, the mean deviations around the X and Y axes were 0.711 and − 0.804 degrees, respectively, with 95% confidence intervals that spanned both positive and negative values. The largest rotational error was found around the Z axis, with a mean of −1.021 degrees. The confidence interval for this component contained exclusively negative values (−1.903 to −0.139), indicating a consistent rotational deviation in this direction across the patient cohort. For the translational components, the mean errors were all small in magnitude: 0.354 mm (X axis), −0.378 mm (Y axis), and − 0.396 mm (Z axis). The confidence intervals for all three translational components also spanned both positive and negative values, reflecting variability in the direction of the error across the patient sample. The detailed raw data for each individual measurement are reported in Supplementary Table S1, while a graphical summary of the statistical analysis for each roto-translational component is provided in Supplementary Figure S1.

**Table 2:** Results obtained with the GPL method.

RTM components	
Rot-X	0.711
	(−0.619, 2.041)
Rot-Y	−0.804
	(−1.981, 0.374)
Rot-Z	−1.021
	(−1.903, −0.139)
Trans-X	0.354
	(−0.274, 0.982)
Trans-Y	−0.378
	(−1.002, 0.245)
Trans-Z	−0.396
	(−1.150, 0.358)

The table shows the mean values and 95% confidence intervals. Rotations are expressed in degrees and translations in millimeters.

Reproducibility of the GPL method was also tested by three independent operators on two separate occasions. Due to its fully automated functionality, the GPL method produced identical results regardless of operator or measurement occasion.

### Method A: Landmark-Based analysis

The results for the accuracy and reliability assessment of Method A are presented in Tables 3, 4, and 5. The analysis focused on the A-A’, B-B’, and F-F’ landmark pairs, as these were consistently measurable across all 17 patients. While data for the C-C’ and E-E’ landmark pairs were also collected, they have been excluded as they could only be reliably identified in two patients. Additionally, the distance between the two mandibular lingulae (D-D’ landmark) could not be evaluated in any patient.

**Table 3:** Results for the accuracy measurements obtained through method A by analysing the different landmarks (A-A’, B-B’, F-F’).

	Occasion	O1	O2	O3
A-A’	1	2.581	2.172	2.503
		(1.625, 3.537)	(1.296, 3.049)	(1.578, 3.428)
	2	2.667	2.809	2.620
		(1.349, 3.986)	(1.673, 3.945)	(1.799, 3.441)
B-B’	1	2.075	1.724	2.176
		(1.041, 3.108)	(0.885, 2.564)	(1.264, 3.088)
	2	1.827	2.121	2.134
		(0.863, 2.792)	(1.182, 3.061)	(1.185, 3.083)
F-F’	1	1.063	1.568	1.636
		(0.475, 1.651)	(1.039, 2.097)	(0.919, 2.353)
	2	1.653	2.077	1.824
		(0.942, 2.364)	(1.437, 2.716)	(1.339, 2.308)

Mean values and 95% confidence intervals are reported for each operator on the two analysis occasions (1, 2). All measurements are express in millimeters.

**Table 4:** Intra-operator variability in the accuracy measurements obtained with method A, analysing the different landmarks (A-A’, B-B’, F-F’).

	O1	O2	O3
A-A’	0.086	0.637	0.117
	(−0.563, 0.736)	(−0.227, 1.500)	(−0.549, 0.783)
B-B’	−0.247	0.397	−0.042
	(−0.540, 0.045)	(−0.177, 0.971)	(−0.360, 0.276)
F-F’	0.590	0.509	0.188
	(−0.106, 1.286)	(−0.282, 1.299)	(−0.695, 1.071)

Mean values and 95% confidence intervals are reported for each operator. All measurements are express in millimeters.

**Table 5:** Inter-operator variability in the accuracy measurements obtained with method A.

	Occasion	O1 vs. O2	O1 vs. O3	O2 vs. O3
A-A’	1	−0.505	−0.573	−0.068
		(−1.420, 0.410)	(−1.208, 0.063)	(−1.125, 0.989)
	2	−0.424	−0.171	0.253
		(−1.540, 0.693)	(−0.897, 0.556)	(−0.619, 1.125)
B-B’	1	0.350	−0.102	−0.452
		(−0.306, 1.007)	(−0.631, 0.428)	(−1.123, 0.219)
	2	−0.294	−0.307	−0.013
		(−0.782, 0.194)	(−0.555, −0.059)	(−0.495, 0.469)
F-F’	1	0.408	0.078	−0.331
		(−0.498, 1.315)	(−0.772, 0.927)	(−1.640, 0.978)
	2	−0.142	0.047	0.189
		(−1.099, 0.816)	(−1.023, 1.116)	(−0.479, 0.856)

Mean values and 95% confidence intervals are reported for the two occasions (1, 2). All measurements are expressed in millimeters.

A detailed analysis of the reconstruction error (delta) revealed that the highest degree of accuracy was achieved at the F-F’ landmark, where the mean reconstruction error consistently ranged between 1.0 mm and 2.1 mm across all operators and occasions. Conversely, the lowest degree of accuracy was identified at the A-A’ landmark where the mean reconstruction error was consistently the largest, typically exceeding 2.5 mm and reaching up to 2.8 mm.

The analysis of intra-operator reliability, summarized in Table 4, revealed that measurement consistency varied considerably depending on the landmark pair. The highest consistency was observed for the B-B’ landmark, where the mean differences between sessions were small (ranging from − 0.247 mm to 0.397 mm) and the confidence intervals were the narrowest. In contrast, the F-F’ landmark and the A-A’ landmark showed lower precision. For these pairs, the mean differences were larger, reaching up to 0.637 mm, and the confidence intervals were substantially wider, in some cases spanning a range of over 1.7 mm (e.g., −0.227 to 1.500 mm for A-A’).

The inter-operator reliability analysis, summarized in Table 5, revealed evidence of operator-dependent variability. The most notable finding was for the B-B’ landmark pair during the second measurement occasion. Here, the comparison between Operator 1 and Operator 3 yielded a mean difference of −0.307 mm, with a 95% confidence interval ranging from − 0.555 to −0.059 mm. The exclusively negative range of this interval confirms a systematic disagreement between the two operators. For the other landmark comparisons, while numerical differences were present, their 95% confidence intervals were wide and spanned both positive and negative values, indicating a lack of precision and consensus among the operators for those measurements.

The detailed raw data for each measurement are reported in Supplementary Table S2, while a graphical summary of the statistical analysis for each landmark is provided in Supplementary Figures S2 (A, B, C).

### Method B: Surface-based analysis

The results for the accuracy and reliability assessment of Method B are presented in Tables 6 and 7, and 8. The analysis focused on the mean (Hmean) and maximum (Hmax) Hausdorff distances; the minimum Hausdorff distance was excluded as it was consistently 0 mm, and the Root Mean Square (RMS) data were not analysed independently as the RMS value is a measure of variance directly associated with the mean.

**Table 6:** Results for the analysis obtained with method B, describing the maximum and mean hausdorff distance.

	Occasion	O1	O2	O3
Hmax	1	10.124	10.160	10.123
		(7.348, 12.900)	(7.430, 12.890)	(7.398, 12.849)
	2	9.931	10.186	9.433
		(7.191, 12.671)	(7.403, 12.968)	(6.836, 12.031)
Hmean	1	0.824	0.810	0.807
		(0.630, 1.018)	(0.607, 1.013)	(0.615, 0.998)
	2	0.823	0.783	0.763
		(0.624, 1.021)	(0.602, 0.964)	(0.585, 0.940)

Mean values and 95% confidence intervals are reported for each operator on the two analysis occasions (1, 2). All measurements are expressed in millimeters.

**Table 7:** Intra-operator variability in the analyses obtained through method B, describing the maximum (Maximum) and mean (Mean) hausdorff distance.

	O1	O2	O3
Hmax	−0.193	0.025	−0.690
	(−0.415, 0.029)	(−0.158, 0.209)	(−1.928, 0.549)
Hmean	−0.002	−0.027	−0.044
	(−0.034, 0.031)	(−0.084, 0.030)	(−0.132, 0.043)

Mean values and 95% confidence intervals are reported for each operator. All measurements are expressed in millimeters.

**Table 8:** Inter-operator variability in the analyses obtained with method B, describing the maximum (Maximum) and mean (Mean) hausdorff distance.

	Occasion	O1 vs. O2	O1 vs. O3	O2 vs. O3
Hmax	1	−0.036	0.001	0.037
		(−0.431, 0.359)	(−0.295, 0.298)	(−0.293, 0.367)
	2	−0.254	0.498	0.752
		(−0.710, 0.201)	(−1.015, 2.011)	(−1.069, 2.574)
Hmean	1	0.015	0.017	0.003
		(−0.034, 0.063)	(−0.030, 0.065)	(−0.026, 0.031)
	2	0.040	0.060	0.020
		(−0.038, 0.119)	(−0.042, 0.162)	(−0.118, 0.159)

Mean values and 95% confidence intervals are reported. All measurements are expressed in millimeters.

The accuracy results for Method B (Table 6) provided two distinct metrics: the mean (Hmean) and maximum (Hmax) Hausdorff distance. A notable difference in magnitude was observed between these two values. The mean Hausdorff distance (Hmean) indicated a low global deviation, with values consistently around 0.8 mm across all operators and occasions. In contrast, the maximum Hausdorff distance (Hmax) was substantially higher, with values around 10 mm, indicating the presence of larger, localized points of deviation.

The analysis of intra-operator reliability (Table 7) for Method B demonstrated excellent consistency, particularly for the mean Hausdorff distance (Hmean). The mean differences between measurement occasions for Hmean were negligible for all operators, with values as low as −0.002 mm. The narrow confidence intervals all included zero, confirming an absence of systematic bias and very high reproducibility for this metric. The maximum Hausdorff distance (Hmax) showed slightly more variability, although it was not statistically significant.

The analysis of inter-operator reliability (Table 8) showed that the maximum Hausdorff distance (Hmax) had larger numerical differences between operators, particularly during the second occasion, indicating less agreement for this specific metric. In stark contrast, the reliability for the mean Hausdorff distance (Hmean) was high. In the first measurement occasion, the agreement was nearly perfect, with mean differences between operators consistently below 0.02 mm and confidence intervals as narrow as 0.057 mm in range. During the second measurement occasion, the inter-operator variability for the Hmean metric increased slightly. The mean differences between operators rose to a maximum of 0.060 mm, and the associated confidence intervals widened, indicating a minor decrease in measurement precision. Despite this, the overall agreement for the Hmean metric was maintained, confirming a substantial degree of precision and objectivity.

The detailed raw data for each measurement are reported in Supplementary Table S3, while a graphical summary of the statistical analysis for each landmark is provided in Supplementary Figures S3(A, B).

## Discussion

Ensuring surgical accuracy is critical in computer-assisted mandibular reconstruction to achieve optimal functional and aesthetic outcomes. Quantitative assessment comparing postoperative results with the virtual surgical plan (VSP) is required for quality control and technique refinement. However, the field still lacks a standardized, universally accepted evaluation methodology. Variability in assessment techniques, measurement parameters, and workflows makes it difficult to compare accuracy results across studies and institutions, highlighting the need for robust and objective evaluation protocols^3^.

Within this context of methodological heterogeneity, the present study validated a novel automated method - the Global Positioning Layout (GPL) and compared its performance with two representative techniques from the literature: a manual, landmark-based analysis (Method A) and a semi-automated, surface-based comparison (Method B).

The primary criterion for selecting reference protocols was their potential for broad applicability across all patients undergoing mandibular reconstruction, regardless of surgical technique. Methods requiring linear, angular, or volumetric measurements of individual bone segments were excluded, as they are applicable only to reconstructions with microvascular osseous free flaps^13,14,21–23^. Approaches using extra-mandibular reference frames on the midface were also excluded to avoid non-eliminable sources of imprecision, such as intrinsic mandibular mobility, surgically induced soft-tissue changes, and the lack of a clear and unequivocal definition of patients occlusion during CT acquisition^24^.

The GPL method proved high objectivity, as its automated protocol yielded identical results across different operators and sessions, eliminating inter- and intra-operator variability. The analysis showed high translational accuracy, with mean errors below 0.5 mm on all three axes. Regarding the rotational components, minimal deviations were quantified around the X- and Y-axes, while a consistent rotational deviation was identified around the Z-axis across the patient cohort. This systematic deviation likely stems from a combination of procedural and biomechanical factors, potentially involving the intrinsic tolerances of the cutting guides, the biomechanical seating of the prosthesis upon fixation, and the influence of the surrounding soft tissues. The ability to quantify deviations through their distinct translational and rotational components within a standardized reference system is a major advantage of the GPL method, as it enables a complete spatial assessment of the final construct’s position and orientation.

Although a standardized correlation between technical accuracy metrics and long-term functional outcomes has not yet been fully established^3,13^, the precision achieved by the GPL method compares favourably with currently accepted benchmarks. While linear deviations below 2.0 mm and angular deviations below 3.0°–4.0° are generally regarded as clinically negligible^6,9–11^, the sub-millimetric translational accuracy (< 0.5 mm) and the minimal rotational error (~ 1.0°) obtained in this study fall well within these ranges, suggesting a precision that exceeds current clinical requirements.

Method A showed significant reproducibility limitations compared with GPL. Intra-operator analysis of Method A revealed low precision, evidenced by the wide 95% confidence intervals for repeated measurements which, in some cases, spanned over 1.7 mm. Overall, this indicates that even a single operator struggles to replicate measurements with high precision. Inter-operator analysis further confirmed operator-dependent variability, For the B-B’ landmark, the comparison between two operators yielded a mean difference of −0.307 mm with a 95% confidence interval that did not include zero [−0.555 to −0.059], confirming a systematic bias. In contrast, the automated workflow of the GPL method eliminates these sources of imprecision and bias, demonstrating superior reproducibility. Moreover, Method A could not be applied to all patients due to absent anatomical landmarks after extensive resections, whereas GPL was applicable in all 17 cases. In addition, Method A’s definition of accuracy - based on transverse distances only - excludes angular informations, potentially classifying a reconstruction as accurate despite clinically significant inclinations or malocclusions.

In contrast, the Roto-Translational Matrix (RTM) used in GPL avoids this limitation by providing a complete three-dimensional description of positional discrepancies.

Method B’s reliability analysis showed a marked difference between its two primary metrics. The mean Hausdorff distance (Hmean) was generally reproducible, with minimal inter-operator differences (< 0.06 mm). However, precision was lower for one operator, as reflected by a wider confidence interval (− 0.132 to 0.043 mm). In contrast, the maximum Hausdorff distance (Hmax) showed greater variability, with wider confidence intervals in both intra- and inter-operator comparisons.

While Hmean demonstrated high reproducibility, Method B is not fully automated and it requires manual selection of at least four corresponding points to initiate surface alignment. The GPL method, in contrast, automates the entire process, eliminating manual input during critical steps. A more significant limitation of Method B is that the Hausdorff distance is a non-directional, absolute measure, providing no information about error orientation or location. Although Hmean indicated a small average global error (~ 0.8 mm), Hmax revealed localized errors up to ~ 10 mm, highlighting the risk of underestimating clinically relevant deviations when relying solely on mean values. In contrast, the Roto-Translational Matrix (RTM), with its separate spatial components, provides a more comprehensive and clinically interpretable description of discrepancies between 3D models.

The reproducibility and applicability issues seen in Methods A and B reflect the broader methodological diversity in the literature (Supplementary Table 4). Current accuracy assessment approaches can be grouped into four main categories. The most common are manual, landmark-based methods, (e.g., Method A), which are intuitive but prone to operator variability^5,6,8,10–13^. The second are semi-automated, surface-based comparisons (e.g., Method B), which are semi-automatic but provide only non-directional, global error values that may mask clinically relevant local deviations^15,16^.

The remaining two categories, though technologically advanced, were excluded from our comparative analysis for specific methodological reasons. The third category, represented by a single roto-translational method, could not be directly compared due to insufficient methodological detail for reliable replication. The fourth category includes geometric feature-based approaches^14,22^, which evaluate the internal assembly accuracy of free flap segments rather than the overall position and orientation of the reconstructed mandibular arch. In contrast, this focus on internal assembly differs from the primary aim of the GPL, which assesses the final three-dimensional position and orientation of the entire reconstructed arch; thus, direct comparison would be methodologically inconsistent.

However, the present study is subject to certain limitations. First, the analysis is based on a retrospective cohort, which inherently introduces potential bias; to mitigate this, a consecutive series of patients meeting strict inclusion criteria was selected to ensure sample homogeneity. Second, the sample size was limited to 17 patients. Although this cohort covered representative mandibular defect types, future multi-center trials are necessary to confirm these findings across a broader range of clinical scenarios.

Future work should extend the validation of the GPL method to larger, prospective, and multicenter cohorts, incorporating diverse CAD–CAM reconstruction techniques and free-flap designs. Despite the comprehensive numerical description provided by RTM components, clinical interpretation remains challenging. Translating matrix-derived deviations into clinically intuitive visual outputs will be necessary to help surgeons identify procedural errors, implement corrective strategies, and prevent recurrence. Enhancing the clinical interpretability of GPL outputs is therefore a key priority for future development.

Additionally, correlating GPL-derived accuracy metrics with long-term reconstructive outcomes will be essential to define clinically relevant thresholds for acceptable error margins. Establishing such correlations may guide surgical planning, improve patient-specific device design, and support the creation of evidence-based guidelines for quality control in computer-assisted mandibular reconstruction.

In conclusion, this study validates the Global Positioning Layout (GPL) method as a robust and fully reproducible tool for assessing the accuracy of computer-assisted mandibular reconstruction.

By eliminating inter- and intra-operator variability, overcoming the limitations of missing anatomical landmarks, and providing a comprehensive three-dimensional analysis of both translational and rotational discrepancies, the GPL method demonstrates a clear methodological advantage over established approaches. This ensures the data reliability which is a critical prerequisite for correctly correlating surgical precision with functional outcomes.

## Supplementary Information

Below is the link to the electronic supplementary material.

Supplementary Material 1

## Data Availability

All the data supporting our findings are presented in the paper.
