# Supplementary material for: Automated Global Positioning Layout (GPL) for accuracy assessment in CAD-CAM mandibular reconstruction – Method validation
Source: Sci Rep. 2026 Feb 25;16:9590. doi: 10.1038/s41598-025-30516-1 (PMC13009390; doi:10.1038/s41598-025-30516-1)
Supplement: Supplementary file 1 — Supplementary Material 1 [file 41598_2025_30516_MOESM1_ESM.docx]

# Supplementary material

**Automated Global Positioning Layout (GPL) for accuracy assessment in CAD-CAM Mandibular Reconstruction – Method validation**

Elisa Vargiu^1^, Laura Tognin^2^, Giordana Bettini^3^, Giorgia Menapace^2^, Piero Franco^4^, Giorgia Saia^5^, Giorgio Bedogni^6,7^, Roberto Meneghello^1*^ Alberto Bedogni^5,8*^

^1^ Department of Management and Engineering, University of Padova, Padova, Italy.

^2^ Unit of Maxillo-Facial Surgery, Head and Neck Department, University of Parma, Parma, Italy.

^3^ Maxillofacial Surgery Unit, ‘‘S. Anna’’ Hospital, Como, Italy.

^4^ Department of Clinical Orthopaedics, University of Florence, A.O.U Careggi CTO Florence, Italy.

^5^ Department of Neuroscience, Unit of Maxillofacial Surgery, University of Padova, Padova, Italy.

^6^ Department of Medical and Surgical Sciences, Alma Mater Studiorum-University of Bologna, Bologna, Italy.

^7^ Department of Primary Health Care, Internal Medicine Unit addressed to Frailty and Aging, “S. Maria delle Croci” Hospital, AUSL Romagna, Ravenna, Italy.

^8^ Regional Center for the Prevention, Diagnosis, and Treatment of Medication and Radiation-related Bone Diseases of the Head and Neck, Hospital Trust of Padova, Padova, Italy.

* Roberto Meneghello and Alberto Bedogni equally contributed to this work.

**Table S1.** Accuracy results based on roto-translation matrix, obtained with the GPL method.

| **ID patient** | **Matrix side** | **Rot-X** | **Rot-Y** | **Rot-Z** | **Trans-X** | **Trans-Y** | **Trans-Z** |
| --- | --- | --- | --- | --- | --- | --- | --- |
| 1 | Left | -1.449 | 0.881 | -0.571 | 0.602 | 0.159 | -1.165 |
| 1 | Right | 2.373 | 2.071 | 1.695 | 0.171 | 2.589 | -2.896 |
| 2 | Right | 0.972 | -1.257 | -1.147 | 0.840 | 0.158 | 0.094 |
| 3 | Right | 0.410 | -4.199 | -0.959 | 0.496 | -2.730 | 0.247 |
| 4 | Left | 0.308 | -0.830 | -4.145 | -1.916 | 3.904 | -1.502 |
| 4 | Right | -0.751 | -0.144 | -2.726 | 1.371 | -0.679 | -0.557 |
| 5 | Left | 3.993 | -0.819 | 0.189 | 1.089 | 2.789 | -2.765 |
| 5 | Right | 2.037 | -0.105 | -0.920 | -0.328 | -0.467 | 0.327 |
| 6 | Right | -2.235 | 1.207 | 0.096 | 0.143 | -0.319 | -2.023 |
| 7 | Right | 5.400 | -4.573 | -2.314 | -1.472 | -2.197 | 1.898 |
| 8 | Right | 0.957 | 0.293 | -3.368 | 2.024 | -0.525 | 0.264 |
| 9 | Right | -0.302 | 0.085 | -5.040 | 1.825 | 0.710 | -0.584 |
| 10 | Left | 1.423 | -0.165 | 1.561 | 1.629 | 0.323 | -1.155 |
| 11 | Right | -1.831 | 0.739 | -1.928 | 1.248 | -0.090 | -1.384 |
| 12 | Left | -0.887 | -0.606 | -0.247 | 0.373 | -0.325 | -1.046 |
| 13 | Left | 7.667 | -7.550 | -0.221 | -2.860 | -2.570 | 3.915 |
| 14 | Left | -0.126 | 1.247 | 1.318 | 0.287 | 0.206 | 0.173 |
| 15 | Right | -3.101 | 1.995 | -2.518 | 2.184 | 0.728 | -1.970 |
| 16 | Right | -2.376 | -0.280 | -0.597 | 0.196 | 0.250 | -1.871 |
| 17 | Left | 2.855 | -1.126 | 1.245 | -1.381 | -2.456 | 1.033 |

**Table S1**. The third roto-translation matrix, obtained with the GPL method, quantifies the deviations between the preoperative plan and the postoperative outcome. Its rotational components (Rot-X, Rot-Y, Rot-Z) are expressed in degrees, and its translational components (Trans-X, Trans-Y, Trans-Z) are in millimeters.

**Fig. S1.** Analysis of systematic rotation and translation errors


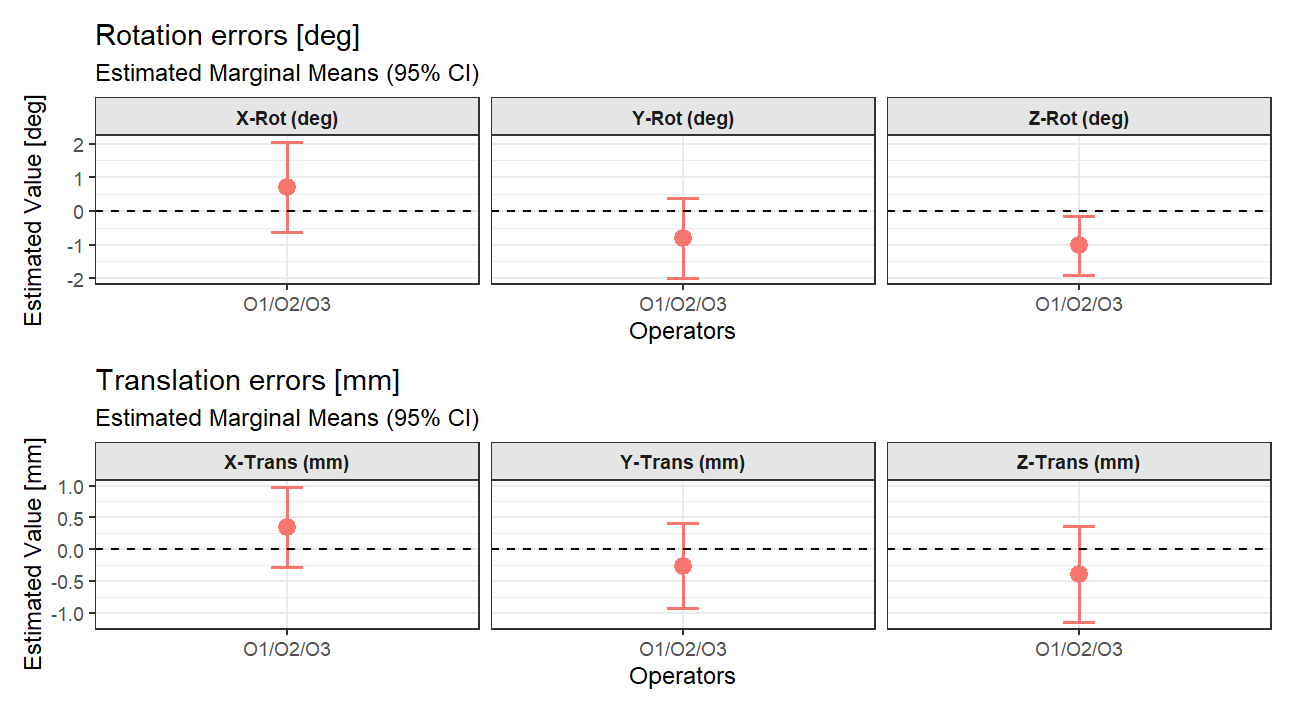


*The figure shows the estimated marginal means (EMMs) with their respective 95% confidence intervals (95% CIs) for the rotation (top) and translation (bottom) errors.*

**Table S2**. Accuracy (Delta) results obtained with method A ^1^.

| **ID patient** | **Occ** | **Checkpoint** | **Delta** | | |
| --- | --- | --- | --- | --- | --- |
|  |  |  | **O1** | **O2** | **O3** |
| 1 | 1 | A-A’ | 2.206 | 1.511 | 2.997 |
|  | 2 | A-A’ | 2.217 | 2.875 | 2.443 |
|  | 1 | B-B’ | 1.996 | 3.399 | 1.520 |
|  | 2 | B-B’ | 1.814 | 1.403 | 1.728 |
|  | 1 | F-F’ | 0.128 | 1.534 | 0.000 |
|  | 2 | F-F’ | 0.825 | 2.439 | 2.637 |
|  | 1 | C-C’ | 3.170 | 2.291 | 1.082 |
|  | 2 | C-C’ | 2.806 | 1.502 | 0.991 |
|  | 1 | E-E’ | 2.799 | 2.420 | 1.751 |
|  | 2 | E-E’ | 1.180 | 1.693 | 2.930 |
| 2 | 1 | A-A’ | 2.799 | 1.730 | 1.536 |
|  | 2 | A-A’ | 1.945 | 0.690 | 1.341 |
|  | 1 | B-B’ | 0.064 | 0.292 | 0.329 |
|  | 2 | B-B’ | 0.133 | 0.796 | 0.787 |
|  | 1 | F-F’ | 1.049 | 1.408 | 0.695 |
|  | 2 | F-F’ | 2.249 | 3.606 | 2.008 |
| 3 | 1 | A-A’ | 0.323 | 2.625 | 0.066 |
|  | 2 | A-A’ | 0.231 | 0.400 | 0.038 |
|  | 1 | B-B’ | 0.085 | 0.022 | 0.187 |
|  | 2 | B-B’ | 0.347 | 0.977 | 0.304 |
|  | 1 | F-F’ | 1.089 | 2.011 | 1.713 |
|  | 2 | F-F’ | 1.019 | 4.231 | 1.126 |
| 4 | 1 | A-A’ | 2.567 | 2.853 | 3.343 |
|  | 2 | A-A’ | 2.404 | 3.446 | 3.140 |
|  | 1 | B-B’ | 0.837 | 0.324 | 2.041 |
|  | 2 | B-B’ | 1.087 | 1.196 | 1.757 |
|  | 1 | F-F’ | 1.000 | 2.211 | 2.345 |
|  | 2 | F-F’ | 1.055 | 4.385 | 2.549 |
| 5 | 1 | A-A’ | 0.216 | 0.030 | 1.095 |
|  | 2 | A-A’ | 0.365 | 0.068 | 1.853 |
|  | 1 | B-B’ | 0.169 | 0.901 | 1.302 |
|  | 2 | B-B’ | 0.249 | 0.786 | 0.439 |
|  | 1 | F-F’ | 2.649 | 0.195 | 1.091 |
|  | 2 | F-F’ | 0.699 | 3.070 | 1.398 |
|  | 1 | C-C’ | 0.015 | 0.247 | 0.096 |
|  | 2 | C-C’ | 0.502 | 0.133 | 0.681 |
|  | 1 | E-E’ | 0.458 | 0.771 | 0.056 |
|  | 2 | E-E’ | 0.140 | 2.097 | 0.104 |
| 6 | 1 | A-A’ | 0.965 | 0.296 | 0.947 |
|  | 2 | A-A’ | 1.990 | 0.244 | 0.741 |
|  | 1 | B-B’ | 0.584 | 0.217 | 1.189 |
|  | 2 | B-B’ | 0.676 | 0.967 | 0.465 |
|  | 1 | F-F’ | 0.221 | 0.632 | 0.535 |
|  | 2 | F-F’ | 0.709 | 0.636 | 0.792 |
| 7 | 1 | A-A’ | 0.859 | 0.665 | 1.369 |
|  | 2 | A-A’ | 0.052 | 1.083 | 1.971 |
|  | 1 | B-B’ | 1.631 | 0.841 | 1.104 |
|  | 2 | B-B’ | 0.282 | 0.950 | 0.997 |
|  | 1 | F-F’ | 0.816 | 1.470 | 1.709 |
|  | 2 | F-F’ | 1.167 | 0.340 | 1.339 |
| 8 | 1 | A-A’ | 3.903 | 4.025 | 3.847 |
|  | 2 | A-A’ | 4.105 | 5.700 | 3.518 |
|  | 1 | B-B’ | 4.944 | 5.456 | 4.785 |
|  | 2 | B-B’ | 4.500 | 5.539 | 4.229 |
|  | 1 | F-F’ | 4.598 | 2.405 | 4.827 |
|  | 2 | F-F’ | 5.667 | 1.739 | 3.570 |
| 9 | 1 | A-A’ | 5.283 | 5.639 | 5.218 |
|  | 2 | A-A’ | 6.176 | 6.305 | 5.373 |
|  | 1 | B-B’ | 6.281 | 3.926 | 4.046 |
|  | 2 | B-B’ | 4.942 | 4.830 | 5.601 |
|  | 1 | F-F’ | 0.330 | 2.779 | 0.629 |
|  | 2 | F-F’ | 5.042 | 3.389 | 3.367 |
| 10 | 1 | A-A’ | 1.778 | 1.026 | 2.135 |
|  | 2 | A-A’ | 0.090 | 2.000 | 1.551 |
|  | 1 | B-B’ | 1.154 | 0.186 | 0.074 |
|  | 2 | B-B’ | 0.028 | 0.238 | 0.200 |
|  | 1 | F-F’ | 1.171 | 0.626 | 4.643 |
|  | 2 | F-F’ | 0.970 | 0.959 | 0.138 |
| 11 | 1 | A-A’ | 5.273 | 5.029 | 0.514 |
|  | 2 | A-A’ | 9.557 | 5.044 | 4.292 |
|  | 1 | B-B’ | 2.613 | 1.983 | 2.770 |
|  | 2 | B-B’ | 2.424 | 1.861 | 2.819 |
|  | 1 | F-F’ | 0.621 | 2.591 | 1.778 |
|  | 2 | F-F’ | 1.174 | 1.896 | 3.607 |
| 12 | 1 | A-A’ | 0.637 | 1.924 | 1.727 |
|  | 2 | A-A’ | 0.111 | 0.384 | 0.774 |
|  | 1 | B-B’ | 0.909 | 1.223 | 1.840 |
|  | 2 | B-B’ | 0.932 | 0.288 | 1.519 |
|  | 1 | F-F’ | 0.194 | 0.100 | 0.543 |
|  | 2 | F-F’ | 0.739 | 0.208 | 1.234 |
| 13 | 1 | A-A’ | 5.221 | 0.465 | 6.405 |
|  | 2 | A-A’ | 5.394 | 5.870 | 5.227 |
|  | 1 | B-B’ | 6.080 | 3.412 | 6.445 |
|  | 2 | B-B’ | 6.123 | 5.744 | 6.266 |
|  | 1 | F-F’ | 0.698 | 0.524 | 1.515 |
|  | 2 | F-F’ | 0.434 | 0.703 | 1.295 |
| 14 | 1 | A-A’ | 0.225 | 0.715 | 0.177 |
|  | 2 | A-A’ | 0.270 | 0.808 | 2.832 |
|  | 1 | B-B’ | 0.068 | 0.418 | 0.192 |
|  | 2 | B-B’ | 0.519 | 0.671 | 1.168 |
|  | 1 | F-F’ | 0.468 | 3.775 | 0.335 |
|  | 2 | F-F’ | 1.255 | 0.838 | 1.458 |
| 15 | 1 | A-A’ | 5.916 | 5.132 | 5.387 |
|  | 2 | A-A’ | 6.000 | 6.449 | 5.432 |
|  | 1 | B-B’ | 4.741 | 4.659 | 4.870 |
|  | 2 | B-B’ | 4.871 | 4.296 | 4.760 |
|  | 1 | F-F’ | 2.990 | 2.995 | 4.061 |
|  | 2 | F-F’ | 2.375 | 1.977 | 2.230 |
| 16 | 1 | A-A’ | 1.479 | 0.339 | 1.495 |
|  | 2 | A-A’ | 0.795 | 1.771 | 0.709 |
|  | 1 | B-B’ | 0.158 | 0.359 | 0.810 |
|  | 2 | B-B’ | 0.010 | 0.910 | 0.043 |
|  | 1 | F-F’ | 0.030 | 1.256 | 0.786 |
|  | 2 | F-F’ | 1.864 | 2.719 | 0.933 |
| 17 | 1 | A-A’ | 4.225 | 2.928 | 4.299 |
|  | 2 | A-A’ | 3.642 | 4.166 | 3.311 |
|  | 1 | B-B’ | 2.953 | 1.697 | 3.493 |
|  | 2 | B-B’ | 2.124 | 4.609 | 3.193 |
|  | 1 | F-F’ | 0.018 | 0.137 | 0.602 |
|  | 2 | F-F’ | 0.858 | 2.168 | 1.319 |

The values represent the difference between the mean distance in the virtual surgical plan (VSP) and the corresponding mean postoperative distance, with measurements taken on two different occasions by three different operators. The results are reported for the following landmarks: A-A’ (innermost point of right to left mandibular condyle), B-B’ (outermost point of right to left mandibular condyle), and F-F’ (most caudal point of right to left mandibular angle), which were assessed in all seventeen patients; and C-C’ (lowest point of right to left mandibular notch) and E-E’ (tip of right to left coronoid process), which were assessed in two patients (patients 1 and 5). All values are expressed in millimeters.

**Fig. S2A**. Repeatability and reproducibility analysis of measurements obtained with method A ^1^


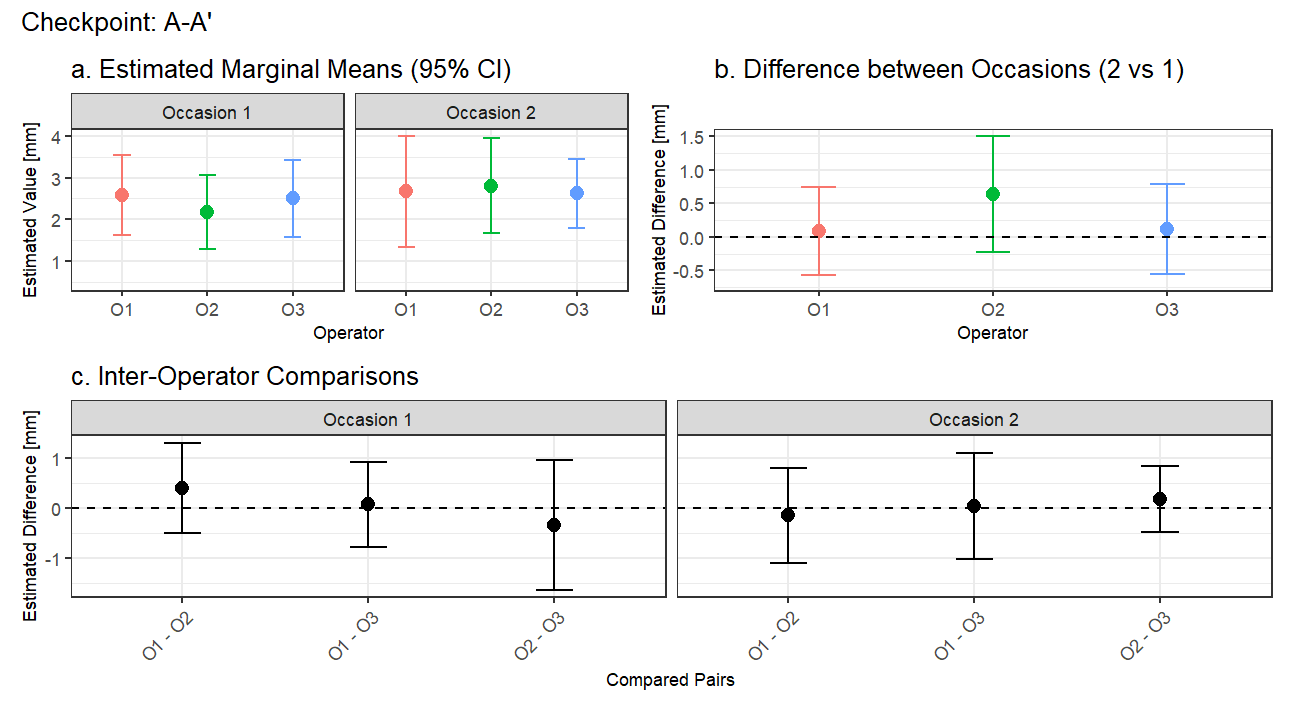


a) Estimated means by operator and occasion. b) Difference between the two occasions per operator (intra-operator repeatability). c) Comparisons between operators for each occasion (inter-operator reproducibility). Error bars indicate 95% confidence intervals.

**Fig. S2B**. Repeatability and reproducibility analysis of measurements obtained with method A^1^ for the B-B' checkpoint.


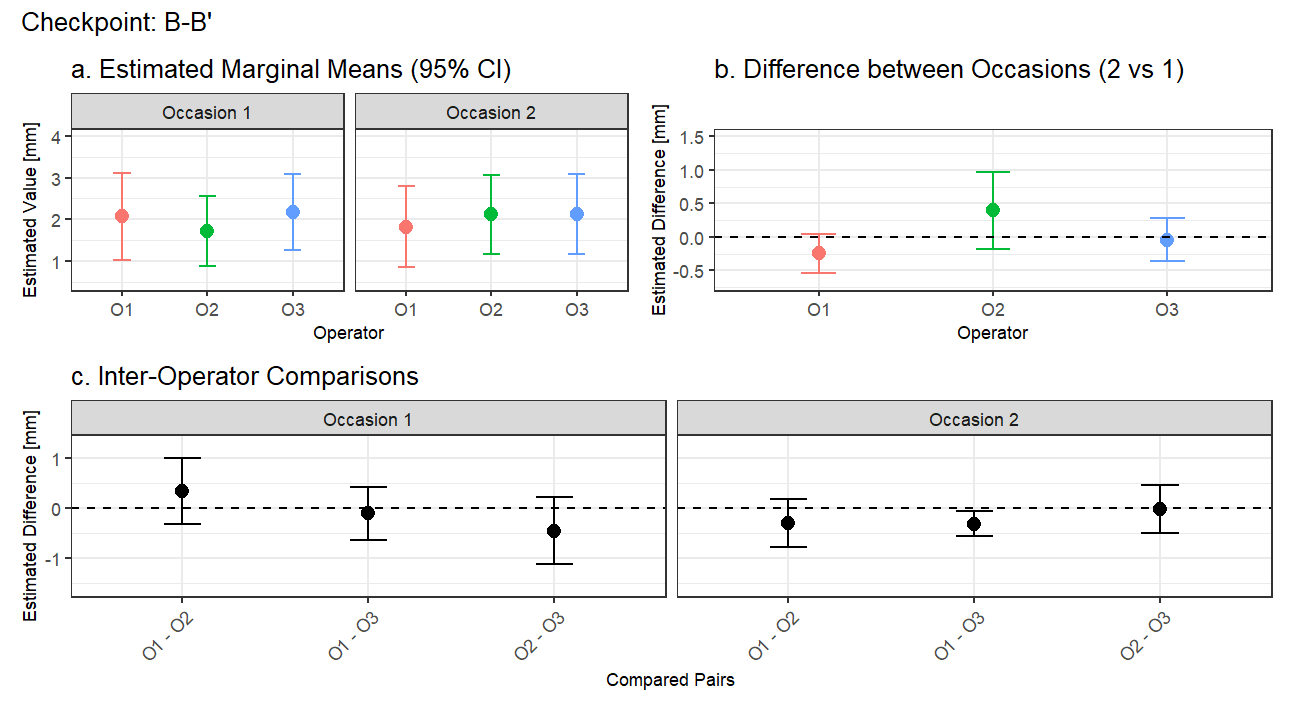


a) Estimated means by operator and occasion. b) Difference between the two occasions per operator (intra-operator repeatability). c) Comparisons between operators for each occasion (inter-operator reproducibility). Error bars indicate 95% confidence intervals.

**Fig. S2C**. Repeatability and reproducibility analysis of measurements obtained with method A^1^ for the F-F' checkpoint.


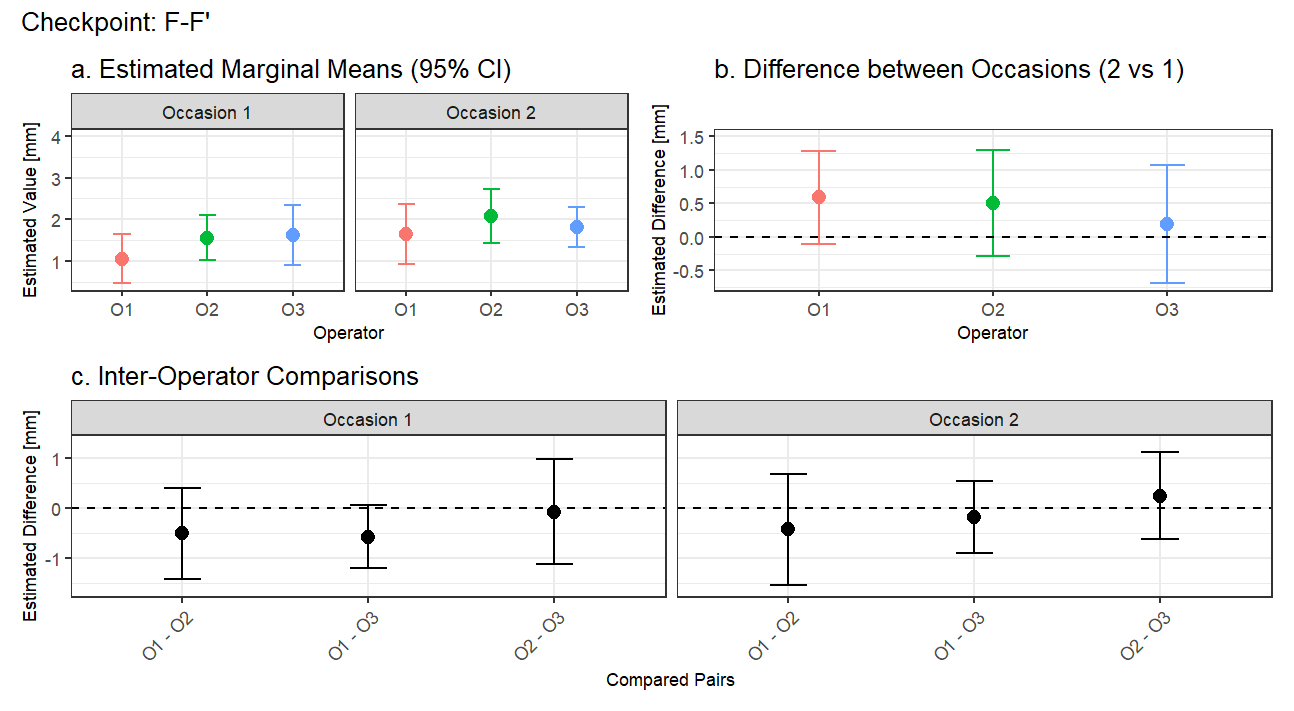


a) Estimated means by operator and occasion. b) Difference between the two occasions per operator (intra-operator repeatability). c) Comparisons between operators for each occasion (inter-operator reproducibility). Error bars indicate 95% confidence intervals.

**Table S3**. Accuracy results based on the Hausdorff distance, obtained with method B^2^**.**

| ID patient | Occ | Distance | O1 | O2 | O3 |
| --- | --- | --- | --- | --- | --- |
| 1 | 1  2 | Hmin | 0.000  0.000 | 0.000  0.000 | 0.000  0.000 |
|  | 1  2 | Hmax | 5.796  4.757 | 5.521  5.695 | 5.687  4.842 |
|  | 1  2 | Hmean | 0.409  0.390 | 0.382  0.381 | 0.383  0.386 |
|  | 1  2 | Hrms | 0.621  0.634 | 0.550  0.571 | 0.569  0.623 |
| 2 | 1  2 | Hmin | 0.000  0.000 | 0.000  0.000 | 0.000  0.000 |
|  | 1  2 | Hmax | 12.002  10.879 | 10.878  10.372 | 10.754  11.181 |
|  | 1  2 | Hmean | 1.036  0.843 | 0.841  0.893 | 0.832  0.848 |
|  | 1  2 | Hrms | 1.850  1.649 | 1.645  1.664 | 1.625  1.677 |
| 3 | 1  2 | Hmin | 0.000  0.000 | 0.000  0.000 | 0.000  0.000 |
|  | 1  2 | Hmax | 13.887  13.205 | 13.716  13.745 | 13.728  13.972 |
|  | 1  2 | Hmean | 0.876  0.968 | 0.872  0.871 | 0.874  0.876 |
|  | 1  2 | Hrms | 1.744  1.713 | 1.716  1.718 | 1.706  1.749 |
| 4 | 1  2 | Hmin | 0.000  0.000 | 0.000  0.000 | 0.000  0.000 |
|  | 1  2 | Hmax | 6.937  7.000 | 6.888  7.081 | 7.018  7.019 |
|  | 1  2 | Hmean | 0.810  0.820 | 0.822  0.803 | 0.799  0.807 |
|  | 1  2 | Hrms | 1.173  1.197 | 1.206  1.170 | 1.156  1.176 |
| 5 | 1  2 | Hmin | 0.000  0.000 | 0.000  0.000 | 0.000  0.000 |
|  | 1  2 | Hmax | 16.683  16.672 | 16.448  16.801 | 16.678  16.764 |
|  | 1  2 | Hmean | 0.812  0.810 | 0.810  0.859 | 0.814  0.826 |
|  | 1  2 | Hrms | 2.249  2.251 | 2.194  2.274 | 2.252  2.277 |
| 6 | 1  2 | Hmin | 0.000  0.000 | 0.000  0.000 | 0.000  0.000 |
|  | 1  2 | Hmax | 8.036  7.905 | 7.938  7.926 | 7.549  7.538 |
|  | 1  2 | Hmean | 0.457  0.457 | 0.455  0.456 | 0.508  0.497 |
|  | 1  2 | Hrms | 0.846  0.833 | 0.834  0.831 | 0.873  0.854 |
| 7 | 1  2 | Hmin | 0.000  0.000 | 0.000  0.000 | 0.000  0.000 |
|  | 1  2 | Hmax | 18.788  18.820 | 18.038  18.117 | 18.807  18.884 |
|  | 1  2 | Hmean | 1.068  1.103 | 1.059  1.045 | 1.076  1.073 |
|  | 1  2 | Hrms | 2.495  2.513 | 2.378  2.388 | 2.506  2.517 |
| 8 | 1  2 | Hmin | 0.000  0.000 | 0.000  0.000 | 0.000  0.000 |
|  | 1  2 | Hmax | 5.108  5.168 | 5.130  5.312 | 5.164  5.130 |
|  | 1  2 | Hmean | 0.631  0.642 | 0.639  0.656 | 0.639  0.638 |
|  | 1  2 | Hrms | 1.194  1.200 | 1.203  1.220 | 1.192  1.206 |
| 9 | 1  2 | Hmin | 0.000  0.000 | 0.000  0.000 | 0.000  0.000 |
|  | 1  2 | Hmax | 15.005  14.959 | 14.824  15.520 | 15.157  15.134 |
|  | 1  2 | Hmean | 1.724  1.826 | 1.806  1.367 | 1.688  1.758 |
|  | 1  2 | Hrms | 2.754  2.891 | 2.835  2.528 | 2.718  0.569 |
| 10 | 1  2 | Hmin | 0.000  0.000 | 0.000  0.000 | 0.000  0.000 |
|  | 1  2 | Hmax | 5.087  5.021 | 5.263  5.190 | 5.388  4.863 |
|  | 1  2 | Hmean | 0.302  0.302 | 0.310  0.308 | 0.318  0.311 |
|  | 1  2 | Hrms | 0.569  0.566 | 0.594  0.581 | 0.599  0.569 |
| 11 | 1  2 | Hmin | 0.000  0.000 | 0.000  0.000 | 0.000  0.000 |
|  | 1  2 | Hmax | 7.351  7.172 | 7.552  7.056 | 7.423  7.242 |
|  | 1  2 | Hmean | 0.857  0.849 | 0.853  0.863 | 0.846  0.840 |
|  | 1  2 | Hrms | 1.336  1.325 | 1.326  1.343 | 1.318  1.312 |
| 12 | 1  2 | Hmin | 0.000  0.000 | 0.000  0.000 | 0.000  0.000 |
|  | 1  2 | Hmax | 2.541  2.494 | 2.511  2.638 | 2.705  2.653 |
|  | 1  2 | Hmean | 0.303  0.307 | 0.303  0.289 | 0.289  0.287 |
|  | 1  2 | Hrms | 0.407  0.412 | 0.407  0.389 | 0.389  0.386 |
| 13 | 1  2 | Hmin | 0.000  0.000 | 0.000  0.000 | 0.000  0.000 |
|  | 1  2 | Hmax | 18.613  17.894 | 19.625  19.932 | 18.025  8.065 |
|  | 1  2 | Hmean | 1.513  1.475 | 1.575  1.593 | 1.489  0.801 |
|  | 1  2 | Hrms | 2.934  2.834 | 3.088  3.137 | 2.849  1.279 |
| 14 | 1 | Hmin | 0.000 | 0.000 | 0.000 |
|  | 2 |  | 0.000 | 0.000 | 0.000 |
|  | 1  2 | Hmax | 2.792  3.046 | 3.160  2.394 | 3.238  2.820 |
|  | 1  2 | Hmean | 0.369  0.405 | 0.410  0.310 | 0.431  0.329 |
|  | 1  2 | Hrms | 0.508  0.574 | 0.583  0.417 | 0.623  1.569 |
| 15 | 1  2 | Hmin | 0.000  0.000 | 0.000  0.000 | 0.000  0.000 |
|  | 1  2 | Hmax | 7.607  7.564 | 7.575  7.735 | 7.529  7.672 |
|  | 1  2 | Hmean | 0.880  0.871 | 0.879  0.886 | 0.869  0.877 |
|  | 1  2 | Hrms | 1.566  1.552 | 1.572  1.576 | 1.550  1.569 |
| 16 | 1  2 | Hmin | 0.000  0.000 | 0.000  0.000 | 0.000  0.000 |
|  | 1  2 | Hmax | 6.268  6.655 | 8.033  8.260 | 7.198  6.994 |
|  | 1  2 | Hmean | 0.740  0.688 | 0.519  0.530 | 0.546  0.583 |
|  | 1  2 | Hrms | 1.126  1.036 | 0.774  0.794 | 0.817  0.873 |
| 17 | 1  2 | Hmin | 0.000  0.000 | 0.000  0.000 | 0.000  0.000 |
|  | 1  2 | Hmax | 19.614  19.622 | 19.627  19.384 | 20.044  19.592 |
|  | 1  2 | Hmean | 1.227  1.229 | 1.232  1.196 | 1.316  1.226 |
|  | 1  2 | Hrms | 2.928  2.935 | 2.937  2.855 | 3.103  2.922 |

The table shows the values for minimum distance (Hmin), maximum (Hmax), mean (Hmean), and root mean square (Hrsm). Measurements were taken on two different occasions by three different operators. All values are expressed in millimeters.

**Fig. S3A.** Repeatability and reproducibility analysis of measurements obtained with method B ^2^ for the Maximum distance (Hmax).


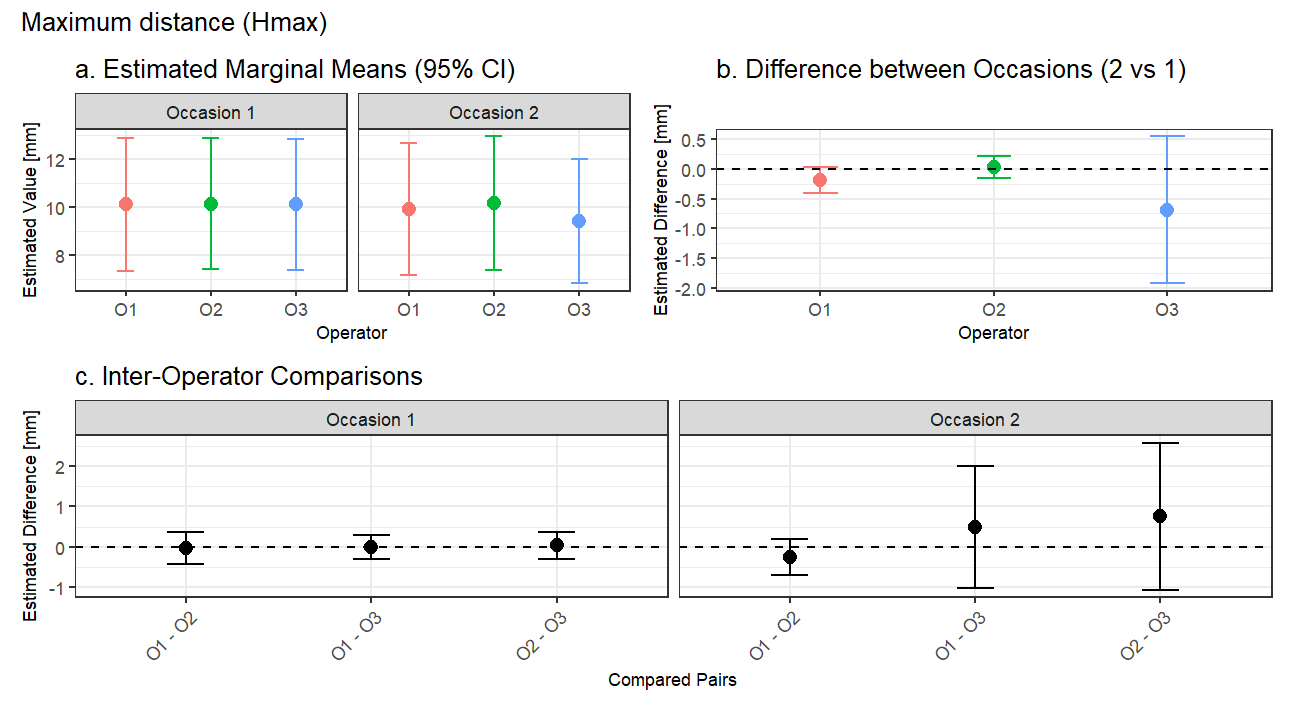


a) Estimated means by operator and occasion. b) Difference between the two occasions per operator (intra-operator repeatability). c) Comparisons between operators for each occasion (inter-operator reproducibility). Error bars indicate 95% confidence intervals.


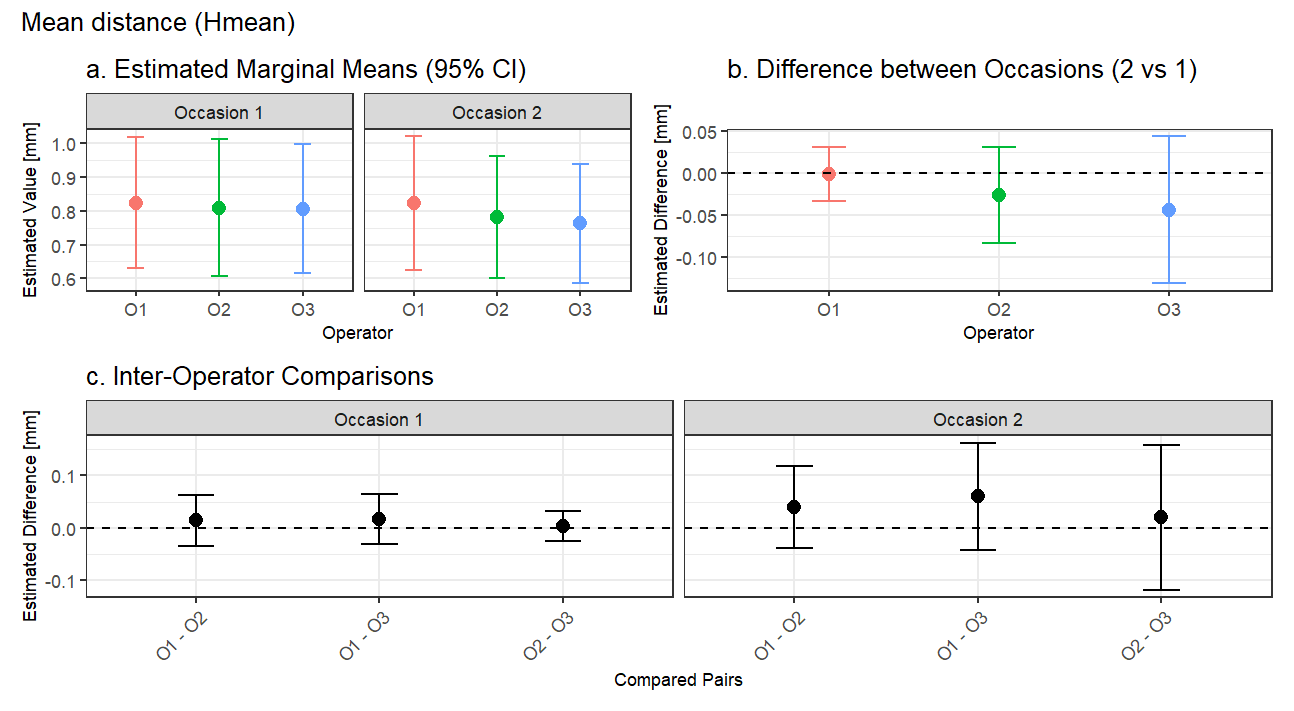
**Fig. S3B**. Repeatability and reproducibility analysis of measurements obtained with method B ^2^ for the Mean distance (Hmean).

a) Estimated means by operator and occasion. b) Difference between the two occasions per operator (intra-operator repeatability). c) Comparisons between operators for each occasion (inter-operator reproducibility). Error bars indicate 95% confidence intervals.

***Table S4****: Summary of accuracy assessment methodologies in mandibular reconstruction.*

| **Authors** | **Method**  (preop-postop) | **Core methodology** | **Measurements** | **Operator input** | **N° of patients** | **Defect's type**  (Anatomical location) | **Reliability assessment** |
| --- | --- | --- | --- | --- | --- | --- | --- |
| Roser et al., 2010 ^3^ | Hybrid surface and morphometric (after 3D superimposition) analysis | Landmark and surface-based | - Fibular bone volume - Linear deviation of osteotomies - Plate contour (% of surface overlap) - Plate position (max distance) | Manual | 11 | NR | NR |
| Foley et al., 2013 ^4^ | Morphometric (after 3D superimposition) analysis | Landmark-based | - Intercondylar distance - Intergonial distance - Anterior-posterior distance | Manual | 8 | - Body (3, including 1 bilateral) - Symphysis (1) - Body and symphysis (4) | NR |
| Metzler et al., 2014 ^5^ | Morphometric analysis | Landmark-based | - Fibula segment dimensions (length, height, width) - Intercondylar distance - Mandibular angle (anterior, posterior) - Condylar angle | Manual | 10 | - Body (4) - Body and ramus (3) - Symphysis and body (2) - Ramus-symphysis-ramus (1) | Inter-operator (2 operators) |
| Wilde et al., 2015 ^1^ | Morphometric analysis | Landmark-based | - A-A': innermost point of right to left condyle - B-B': outermost point of right to left condyle - C-C': lowest point of right to left mandibular notch - D-D': tip of right to left lingula - E-E': tip of right to left coronoid process - F-F': most caudal point of right to left gonial angle | Manual | 36* | NR** | NR |
| Schepers et al., 2015 ^6^ | Geometric feature (after 3D superimposition) analysis | Geometric feature-based | - Linear and angular deviation (based on virtual cylinders for fibula segments and implants) | Semi-automated | 7 | NR | Inter-operator (2 operators in 5 cases) |
| Hanken et al., 2015 ^7^ | Morphometric (after 3D superimposition) analysis | Landmark-based | - Defect size, - Position, angulation, and volume of the transplanted bone segments (fibula or iliac crest) | Manual | 26 | Jewer et al. Classification ^8^:   - LCL (5) - LC (2) - C (4) - L (14) - H (1) | Intra-operator (1 operator on 3 random cases) |
| Zhang et al., 2015 ^9^ | Hybrid surface (after 3D superimposition) and morphometric analysis | Landmark and surface-based | - Linear, angular, and surface deviation (per fibula segment) - Intercondylar distance - Intergonial distance - Gonial angle | Manual | 8 | NR | NR |
| Tarsitano et al.,2018 ^2^ | Surface (after 3D superimposition) analysis | Surface-based | - Global surface deviation (Hausdorff distance: min, max, mean) | Semi-automated | 34 | Tarsitano classification^10^:   - Class I (6) - Class Ic (12) - Class II (9) - Class III (7) | NR |
| Goormans et al., 2019 ^11^ | Morphometric (after 3D superimposition) analysis | Landmark-based | - Length deviation (per fibula segment), - Angular deviation (per osteotomy) - Intercoronoid distance, - Intergonial distance, - Anterior-posterior distance - Intersegmental plane shift | Manual | 26*** | Grouped by complexity:   - Condyle preserved (18) - Condyle resected (8) - 1-segment (8) - 2-segment (11) - 3-segment (7) | NR |
| Zhou et al.,2019 ^12^ | Surface (after 3D superimposition) analysis | Surface-based | - Global surface deviation (part comparison analysis: mean error, std dev) | Manual | 9 | - Lateral mandibular body and partial ramus (condyle preserved) | NR |
| Van Baar et al.,2019 ^13^ | Morphometric (after 3D superimposition) analysis | Landmark-based | - Mandibular angles (coronal, axial, sagittal) - XYZ deviations of dental implants | Manual | 3 | Brown et al. Classification^14^ :   - Class I (1) - Class II (1) - Class III (1) | N/A |
| Geusens et al., 2019 ^15^ | Morphometric analysis | Landmark-based | - Length of fibula segments (superior and inferior borders) - Intercoronoid distance | Manual | 20 | NR | Inter-operator (2 operators) |
| Chernohorskyi et al., 2021 ^16^ | Hybrid surface and morphometric (after 3D superimposition) analysis | Landmark and surface-based | - Global surface deviation (mean and maximum) - Intercondylar distance - Intergonial distance - Gonial angle | Manual | 40 | NR | NR |
| Zavattero et al., 2021 ^17^ | Morphometric (after 3D superimposition) analysis | Landmark-based | - Symphysis - Right condyle - Left condyle - Right mandibular angle - Left mandibular angle | Manual | 47 | NR ** | Intra-operator (1 operator, 3 repetitions) |
| Annino Jr et al., 2022 ^18^ | Morphometric (after 3D superimposition) analysis | Landmark-based | - Intercondylar, intergonial, - Anterior-posterior distance - Gonial angle - Length deviation of bone segments | Manual | 60 | Brown's classification ^14^:   - Class I (34) - Class 1c (2) - Class II (11) - Class III (10) - Class IV (3) | NR |
| El-Mahallawy et al., 2023 ^19^ | Morphometric (after 3D superimposition) analysis | Landmark-based | - Mandibular angles (coronal, axial, sagittal) - Intercondylar distance - Intergonial distance - Anterior-posterior distance | Manual | 9 | Brown's classification ^14^:   - Class I (3) - Class II (6) | Inter-operator (2 operators) |
| Bevini et al., 2023 ^20^ | Hybrid spatial displacement and surface (after 3D superimposition) analysis | Roto-translational and surface-based | - Roto-translational discrepancies (XYZ) rotations and translations for bone stumps - Surface deviation of the neo-alveolar crest | Semi-automated | 10 | NR | NR |
| Bao et al., 2024 ^21^ | Geometric feature (after 3D superimposition) analysis | Geometric feature-based | - Length, angular, and positional deviation (based on inertial axis and center of gravity of segments) | Semi-automated | 35 | Brown's classification ^14^:   - Class I (12) - Class II (18) - Class III (5) | Inter-operator (2 operators) |
| Nahass et al., 2025 ^22^ | User-defined plan analysis | Landmark-based | - Angular deviation between manually defined planes | Manual | 1 | NR | Intra-operator (1 operator, 5 repetitions) |
| Borbon et al., 2025 ^23^ | User-defined plan analysis | Landmark-based | - Angular deviation between osteotomy planes - Thickness of the deviation volume | Manual | 17 | - Body and ramus (7) - Body (5) - Symphysis and body (5) | NR |

Synthesis of the Literature on Accuracy Assessment Methods in Computer-Assisted Mandibular Reconstruction. The table provides a comparative summary of 20 studies detailing the method, the core methodology, the measurements performed, the operator input, the number of patients, the defect types analysed, and the reliability assessment protocols employed.

Legend:

NR: not reported

N/A: not applicable

* Among the 36 patients, multiple procedures in some cases brought the total operations analyzed to 42.

**In these studies, defects are classified by anatomical region. Since a single defect may involve multiple adjacent regions (e.g., body and angle), a patient can appear in more than one category, so the total defects may exceed the number of patients.

*** From the 30 cases analyzed, 4 involving maxillary reconstructions were excluded.

**REFERENCES**

1. Wilde, F. *et al.* Multicenter study on the use of patient-specific CAD/CAM reconstruction plates for mandibular reconstruction. *Int J Comput Assist Radiol Surg* **10**, 2035–2051 (2015).

2. Tarsitano, A. *et al.* Accuracy of CAD/CAM mandibular reconstruction: A three-dimensional, fully virtual outcome evaluation method. *Journal of Cranio-Maxillofacial Surgery* **46**, 1121–1125 (2018).

3. Roser, S. M. *et al.* The Accuracy of Virtual Surgical Planning in Free Fibula Mandibular Reconstruction: Comparison of Planned and Final Results. *Journal of Oral and Maxillofacial Surgery* **68**, 2824–2832 (2010).

4. Foley, B. D., Thayer, W. P., Honeybrook, A., McKenna, S. & Press, S. Mandibular reconstruction using computer-aided design and computer-aided manufacturing: An analysis of surgical results. *Journal of Oral and Maxillofacial Surgery* **71**, (2013).

5. Metzler, P., Geiger, E. J., Alcon, A., Ma, X. & Steinbacher, D. M. Three-dimensional virtual surgery accuracy for free fibula mandibular reconstruction: Planned versus actual results. *Journal of Oral and Maxillofacial Surgery* **72**, 2601–2612 (2014).

6. Schepers, R. H. *et al.* Accuracy of fibula reconstruction using patient-specific CAD/CAM reconstruction plates and dental implants: A new modality for functional reconstruction of mandibular defects. *Journal of Cranio-Maxillofacial Surgery* **43**, 649–657 (2015).

7. Hanken, H. *et al.* Virtual planning of complex head and neck reconstruction results in satisfactory match between real outcomes and virtual models. *Clin Oral Investig* **19**, 647–656 (2015).

8. Jewer, D. D. *et al.* Orofacial and Mandibular Reconstruction with the Iliac Crest Free Flap: A Review of 60 Cases and a New Method of Classification. *Plast Reconstr Surg* **84**, (1989).

9. Zhang, L. *et al.* Evaluation of computer-assisted mandibular reconstruction with vascularized fibular flap compared to conventional surgery. *Oral Surg Oral Med Oral Pathol Oral Radiol* **121**, 139–148 (2016).

10. Tarsitano, A., Del Corso, G., Ciocca, L., Scotti, R. & Marchetti, C. Mandibular reconstructions using computer-aided design/computer-aided manufacturing: A systematic review of a defect-based reconstructive algorithm. *Journal of Cranio-Maxillofacial Surgery* **43**, 1785–1791 (2015).

11. Goormans, F. *et al.* Accuracy of computer-assisted mandibular reconstructions with free fibula flap: Results of a single-center series. *Oral Oncol* **97**, 69–75 (2019).

12. Zhou, Z., Zhao, H., Zhang, S., Zheng, J. & Yang, C. Evaluation of accuracy and sensory outcomes of mandibular reconstruction using computer-assisted surgical simulation. *Journal of Cranio-Maxillofacial Surgery* **47**, 6–14 (2019).

13. van Baar, G. J. C., Liberton, N. P. T. J., Forouzanfar, T., Winters, H. A. H. & Leusink, F. K. J. Accuracy of computer-assisted surgery in mandibular reconstruction: A postoperative evaluation guideline. *Oral Oncol* **88**, 1–8 (2019).

14. Brown, J. S., Barry, C., Ho, M. & Shaw, R. A new classification for mandibular defects after oncological resection. *The Lancet Oncology* vol. 17 e23–e30 Preprint at https://doi.org/10.1016/S1470-2045(15)00310-1 (2016).

15. Geusens, J. *et al.* Accuracy of Computer-Aided Design/Computer-Aided Manufacturing-Assisted Mandibular Reconstruction With a Fibula Free Flap. *J Craniofac Surg* **30**, 2319–2323 (2019).

16. Chernohorskyi, D. M., Chepurnyi, Y. V., Vasiliev, O. S., Voller, M. V. & Kopchak, A. V. Evaluation of the accuracy of surgical reconstruction of mandibular defects when using navigation templates and patient-specific titanium implants. *Journal of Education, Health and Sport* **11**, 117–132 (2021).

17. Zavattero, E. *et al.* Accuracy of Fibula Reconstruction Using Patient-Specific Cad/Cam Plates: A Multicenter Study on 47 Patients. *Laryngoscope* **131**, E2169–E2175 (2021).

18. Annino, D. J. *et al.* Virtual planning and 3D-printed guides for mandibular reconstruction: Factors impacting accuracy. *Laryngoscope Investig Otolaryngol* **7**, 1798–1807 (2022).

19. El-Mahallawy, Y., Abdelrahman, H. H. & Al-Mahalawy, H. Accuracy of virtual surgical planning in mandibular reconstruction: application of a standard and reliable postoperative evaluation methodology. *BMC Oral Health* **23**, 119 (2023).

20. Bevini, M., Vitali, F., Ceccariglia, F., Badiali, G. & Tarsitano, A. Accuracy Evaluation of an Alternative Approach for a CAD-AM Mandibular Reconstruction with a Fibular Free Flap via a Novel Hybrid Roto-Translational and Surface Comparison Analysis. *J Clin Med* **12**, 1938 (2023).

21. Bao, T. *et al.* Reliabilities of three methods used to evaluate computer-assisted mandibular reconstructions using free fibula flaps. *Heliyon* **10**, (2024).

22. Nahass, G. R. *et al.* Mathematical methods for assessing the accuracy of pre-planned and guided surgical osteotomies. *International Journal of Computer Assisted Radiology and Surgery* **20**, 891–900 (2025).

23. Borbon, C. *et al.* Evaluating Osteotomy Accuracy in Mandibular Reconstruction: A Preliminary Study Using Custom Cutting Guides and Virtual Reality. *Diseases* **13**, (2025).
